# Colorectal carcinoma tumour budding and podia formation in the xenograft microenvironment

**DOI:** 10.1371/journal.pone.0186271

**Published:** 2017-10-17

**Authors:** Friedrich Prall, Claudia Maletzki, Maja Hühns, Mathias Krohn, Michael Linnebacher

**Affiliations:** 1 Institute of Pathology, University of Rostock, Rostock, Germany; 2 Department of Surgery, University of Rostock, Rostock, Germany; Columbia University, UNITED STATES

## Abstract

Tumour budding and podia formation are well-appreciated in surgical pathology as an aggressive invasion phenotype of colorectal carcinoma cells that is attained in the microenvironment of the invasive margin. In this study, we addressed how tumour budding and podia formation feature in xenografts. Primary colorectal carcinomas (N = 44) of various molecular types (sporadic standard type, high-degree microsatellite-unstable, CpG island methylator phenotype) were transplanted subcutaneously into T and B cell-deficient NSG mice, making possible immunohistochemistry with routine surgical pathology antibodies. Tumor budding and podia formation were both appreciably present in the xenografts. Quantitative evaluations of cytokeratin immunostains of primaries and their corresponding xenografts showed a reduction of tumour buds in the xenografts. Furthermore, in xenografts tumour cells were completely negative by pSTAT3 immunohistochemistry, indicating absence of cytokine/chemokine signalling, but nuclear β-catenin and SMAD4 immunostainings as read-out of wnt and BMP pathway activation, respectively, were maintained. Carcinoma cells in most xenografts retained immunostaining of at least some nuclei by immunohistochemistry with antibodies against pERK1/2. K-ras/B-raf mutational status did not correlate with tumour budding or podia formation in the xenografts. Our results indicate that tumour budding and podia formation can be modelled by xenografting, and in NSG mice it can be studied with the same immunohistochemical methods as used for primaries in surgical pathology. Dysregulation of wnt and BMP signalling appears to be transferred into the xenograft microenvironment, but not cytokine/chemokine signalling.

## Introduction

Cancerous invasive growth is well recognized as a highly complex process and a challenging topic of study. For colorectal carcinoma, different phenotypes of invasion have been described, namely the expansive *vs*. the infiltrative edge, different degrees of tumour budding, and different degrees of podia formation [1]. They are interpreted as a migratory phenotype of the cancer cells and, as an important prognostic factor, they reflect the aggressiveness of a cancer. As of recent, there is consensus on how to assess tumour budding in surgical specimens [2]. This feature, therefore, now is ready for inclusion into standard surgical pathology reports, underpinning the importance of the issue. While tumour budding and podia can be appreciated with confidence by morphological study, the biology behind is incompletely understood. On principle, growth factors, cytokines, and chemokines secreted by the immune and stromal cells may play a role, and the extracellular matrix, remodelled during tumour growth, may also have an effect on tumour cells' migratory properties. All these factors can be summarized as the microenvironment of the invasive edge, which is extrinsic to the tumour cells. In addition, as an intrinsic factor, the “molecular make-up”of the cancer, which in the case of colorectal cancer is reflected in the molecular classes [3], will not only sustain autonomous cancer cell cycling, but may also lead to different pro-migratory outside-in signalling responses of the cancer cells.

For colorectal carcinoma, dysregulation/activation of wnt-signalling, and of the bone morphogenetic pathway (BMP) are known to be cardinal signalling transduction pathways compromised by genomic aberrations. These signalling pathways are amenable to morphologic study of tumour specimens by β-catenin or SMAD4 immunohistochemistry, respectively: as downstream-effectors in the signalling cascades, when activated, these proteins can be found in the nuclei of the tumour cells [4, 5]. Similarly, cytokine and chemokine receptor activation can be demonstrated by nuclear immunostaining with antibodies against phosphorylated STAT3 (pSTAT3) [6], and signalling along the ras axis by immunohistochemistry against the dually phosphorylated extracellular signal-regulated kinase 1 and 2 (p44/p42 ERK1/2) [7].

Traditionally, morphologic study of colorectal carcinoma invasion is based on tissues from surgical resections. The above mentioned phenotypes of invasion have been delineated in the last decade by this type of study. However, in order to gain insight into cellular mechanisms of colorectal carcinoma invasion, studies of model systems that allow observation of tumours in a different microenvironment could be valuable. To this end, xenografts have several advantages: contrary to cell culture, xenografting is a procedure with fairly high success rates [8], thus allowing comparison of larger series of primaries and their low-passage xenografts; they grow in three dimensions, thus allowing morphological study by routine methods; and if xenografting is done in mice deficient in T and B cells such as NSG mice [9], immunohistochemistry may be expected to work with exactly the same reagents and protocols as used for surgical specimens.

Here we report on a series of colorectal carcinomas and their xenografts in NSG mice that comprise all the major molecular subtypes. We addressed, how tumour budding and podia formation in xenografts compared to its primaries, how immunohistochemical studies would work in this setting, and tested if the expression of downstream-effectors of the cardinal potentially dysregulated signal transduction pathways would be altered in the xenograft microenvironment.

## Materials and methods

### Primary colorectal carcinomas and xenografts

Resection specimens were received fresh from the operating theatre in the Institute of Pathology. Prior written informed consent was obtained from all patients, and all procedures were approved by the Ethics Committee of Rostock University (ref. II HV 43/2004). Small cubes (ca. 3 x 3 x 3 mm) were taken from the invasive margins for subcutaneous xenografting into NSG mice, either immediately, or after cryopreservation [8]. All in vivo procedures were approved by the Committee on the Ethics of Animal Experiments of the University of Rostock (Landesamt für Landwirtschaft, Lebensmittelsicherheit und Fischerei Mecklenburg-Vorpommern; Thierfelder Str. 18, 18059 Rostock, Germany; approval number: LALLF M-V/TSD/7221.3–1.1-015-14). Subcutaneous implantation into both flanks was performed in a total of 44 mice under i.p. Ketamin/Xylazin anesthesia (dose: 90/6 mg/kg body weight). Postoperatively, drinking water was supplied with 8 mg Trimethoprime and 40 mg Sulfamethoxazole per liter to prevent bacterial infections from the engrafted colorectal cancers. Animals were kept in cages at a minimum of two and a maximum of five with food and water ad libitum. Animals and tumour growth were monitored twice weekly. Animals were checked according to a score sheet that addresses tumour size, and the animals' weight, appearance, and behaviour. Animals were euthanized when tumours had grown to a size of 1.5 cm^3^ or if 30 weeks after xenografting had elapsed. According to animal welfare regulations euthanization would have been performed if there had occurred tumour ulceration, motoric impairment, rectal bleeding or diarrhoea, or loss of >15% of animals' initial body weight, but this was not observed in this series. Times from implantations to harvesting the xenografts ranged from 4 to 30 weeks. Euthanasia was done using CO_2_ at an air-exchange rate of 20–25% per minute until breathing stopped, followed by cervical dislocation. One half of each xenograft was immersion fixed in buffered formalin (10%) immediately upon removal and processed for histology by routine methods.

The remaining surgical resection specimen was fixed overnight in buffered formalin (10%) and processed for a surgical pathology report. For each case, one of the archived paraffin-blocks of the primary tumour that included representative regions of the invasive margin was selected for this study.

A total of 44 tumours were included in this study. Tumours were classified according to molecular features as sporadic standard type (spSTD; N = 12), sporadic microsatellite-unstable (spMSI-H; N = 12), CpG island methylator phenotype (CIMP; N = 15), or of the Hereditary Non-Polyposis Carcinoma Coli (HNPCC) Syndrome type (N = 5). Detailed information on the molecular studies can be found in [3].

### Immunohistochemical reactions and evaluations

Primary tumours and xenografts were studied by immunohistochemistry with antibodies against cytokeratin (CK) 18, β-catenin, SMAD4, pSTAT3, and the dually phosphorylated extracellular signal-regulated kinase 1 and 2 (pERK1/2) using an autostainer (DAKO autolink 48; Dako, Glostrup, Denmark) with DAB as chromogen (details in Table 1). Nuclear β-catenin translocation was scored as follows: score 2 if nuclear (and cytoplasmic) instead of/in addition to membranous immunostaining was found in ≥5% of the tumour cells along the invasive edge; score 1 for cases with <5%; and score 0 for tumours without any nuclear β-catenin translocation. Loss of SMAD4 or pSTAT3 was scored if all tumour cells remained completely negative (provided stromal cells were positive as internal controls).

**Table 1:** List of antibodies and details of the immunohistochemical reactions.

Antigen	Supplier	Species	Clone	Titre
CK 18	DAKO, Glostrup, DK	mouse monoclonal	DC10	1: 100
β-catenin	DAKO	mouse monoclonal	β-Catenin 1	ready-to-use
SMAD4	Santa Cruz Biotechnology, Heidelberg, Germany	mouse monoclonal	B8	1: 100
pSTAT3	Santa Cruz Biotechnology	mouse monoclonal	B7	1: 100
pERK1/2	CST, Leiden, The Netherlands	rabbit polyclonal	NA	1: 100

### Quantitative evaluations and morphometric studies

Counting of tumour buds and podia was done with the imageJ cell counter tool (freeware available at http://rsb.info.nih.gov/nih-image) on digital microphotos of CK18 immunostains. Areas with maximum tumour budding or podia formation ("hot spots") at the invasive margin of the primaries and the xenografts were selected at scanning magnification and microphotographs covering an area of 0.292 mm^2^ (20x objective) were taken. Tumour buds were defined as CK18 positive cancer cells that, singly or in aggregates of up to four cells, were found discontinuous from the bulk of the tumours. Podia were recorded as a-nucleate CK18 positive globules or “wisps”with diameters of 0.5–5 μm.

### Statistics

Statistics were done with SPSS (SPSS, version 13.0, Chicago, IL, USA). Because the distributions of tumour budding and podia data were skewed (i.e. non-normal, by Kolmogorov-Smirnov test), comparisons of between groups were done by the Mann-Whitney test.

## Results

All immunohistochemical reactions on the paraffin-embedded xenograft tissues were straightforward to perform with the same primary antibodies and secondary reagents used in routine immunohistochemistry of human tumour tissues, producing crisp immunostains without background. Examples of xenograft immunohistochemistry are shown along with the results detailed below.

**Fig 1:**
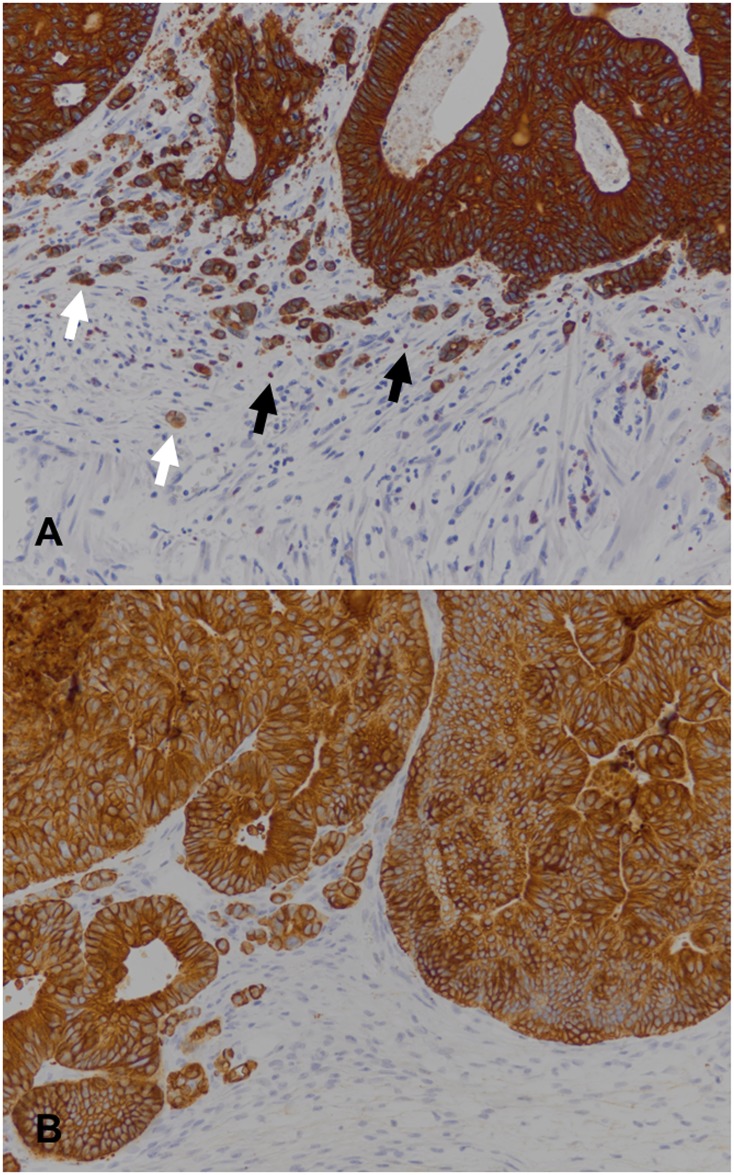
Example of CK18 immunohistochemistry for counting of tumour buds and podia. (A) A primary colorectal carcinoma (HROC39 in Table 2), and (B) its corresponding xenograft. Exemplary tumour buds are denoted with white arrows and podia with black arrows.

**Fig 2:**
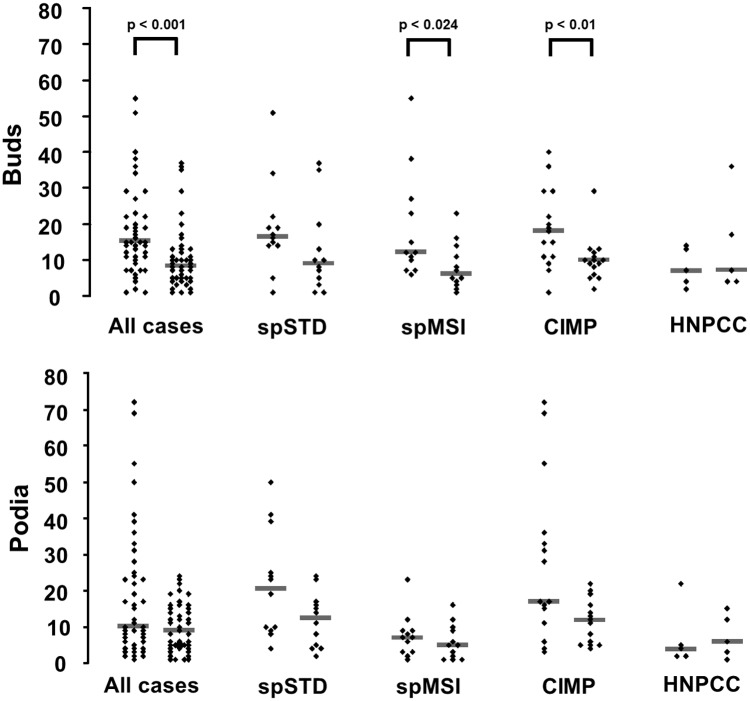
Tumour budding and podia in primary colorectal carcinomas and their corresponding xenografts. Tumour buds and podia were counted on CK18 immunostains in "hot spots" (0.292 mm^2^). Within each group, data obtained from primaries are plotted to the left and data from xenografts are on the right side. Horizontal bars represent medians of the groups. P-values by Mann Whitney test are given where significant.

Counting tumour buds and podia on CK18 immunostains (see Fig 1 for an exemplary image) revealed lower median numbers of both tumour buds and podia in the xenografts as compared to the primaries (data plotted in Fig 2). The differences of medians were statistically significant for tumour budding (Mann-Whitney test p < 0.001), but not quite so for podia (Mann-Whitney test p < 0.057). Stratifying the data by molecular types, statistically significant differences of tumour budding median values were observed for the spMSI and the CIMP cases (Mann-Whitney test p < 0.024 and 0.01, respectively), but not for the other two groups, and not for podia.

**Fig 3:**
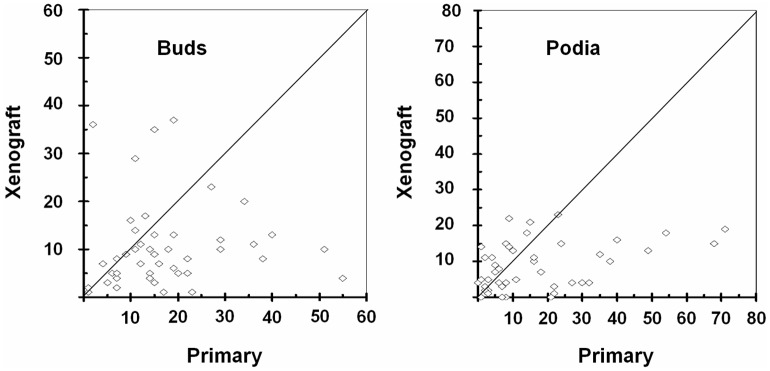
Scatter plots of tumour buds and podia in primaries and their xenografts for case-by-case comparisons. Note that in cases below the diagonal line tumour buds or podia are reduced in xenografts whereas tumour buds or podia in cases above this line are more abundant.

On a case-by-case basis, reduction of tumour buds or podia was not seen universally: in 10 and 16 of the 44 cases, tumour budding or podia, respectively, were more frequent in the xenografts than in the primaries (Fig 3).

**Fig 4:**
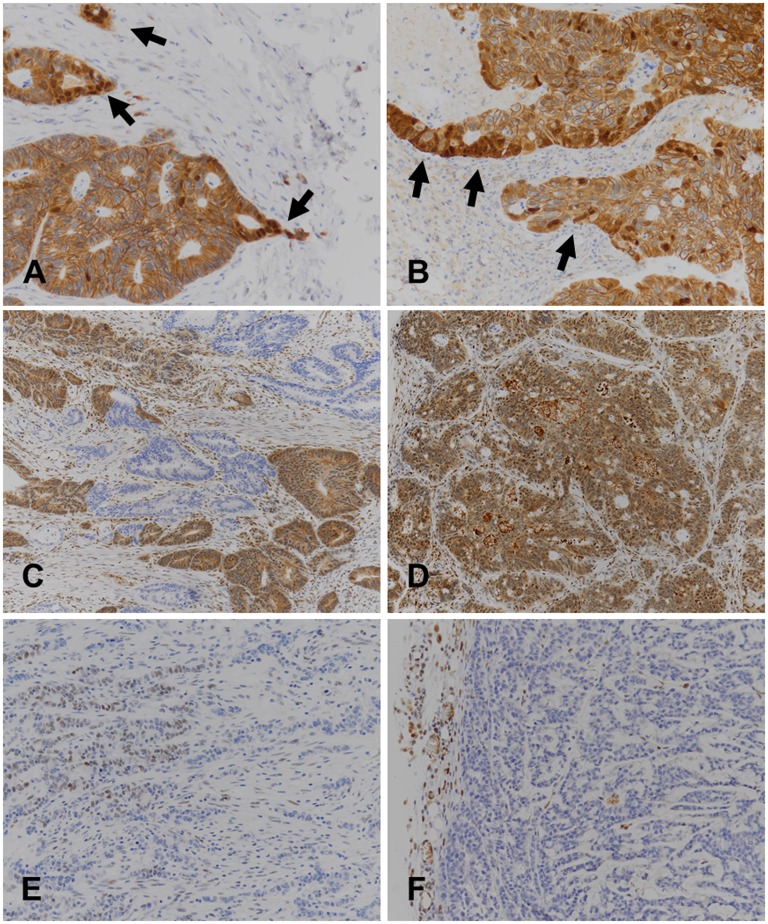
Example of immunohistochemical reactions with primary colorectal carcinomas and xenografts. Images to the left (A, C, E) are from primaries, images to the right (B, D, F) are from their corresponding xenografts. The pair of images at the top (A, B) illustrates how β-catenin nuclear translocation (arrows) is retained in xenografts (tumour HROC192); the pair of images in the middle (C, D) are from SMAD4 immunostains with a peculiar "clonally" negative reaction in the primary (tumour HROC213); and images at the bottom (E, F) show a negative pSTAT3 immunoreaction of the xenografted tumour cells (but positive stromal cell nuclei as internal control), whereas some tumour cell nuclei in the primary are positive (tumour HROC175). Note the crisp immunoreactions without background obtained with the xenografts grown in NSG mice.

In order to explore if tumour cells were subject to cytokine and/or chemokine signalling, we performed pSTAT3 immunohistochemistry. In the xenografts, we observed complete absence of immunostaining in all tumour cells in all cases. Because the pSTAT3 antibody cross-reacts with murine pSTAT3, immunopositive stromal cells were found in all cases, thus providing a convenient internal control for the immunoreactions (example in Fig 4). By contrast, a consistent immunoreaction of stromal cells as internal controls was not seen in pSTAT3 immunohistochemistry of many primaries, precluding their evaluations. However, pSTAT3 immunohistochemistry of primaries for which evaluations were not technically compromised (five cases) revealed immunopositive tumour cell nuclei (example in Fig 4).

Nuclear β-catenin translocation in the primaries was mirrored in the xenografts (see Fig 4 for an example, and Table 2 for overview). In the case of two spMSI tumours (HROC131 and HROC170) which showed a clonal pattern of β-catenin immunohistochemistry, the pattern dominating the primary was seen in the xenografts.

**Table 2:** List of cases included in the study, molecular features, and results of β-catenin and SMAD4 immunohistochemistry.

Tumour ID	Molecular type	K-Ras	B-Raf	β-Catenin Score		SMAD4	
				Primary	Xeno	Primary	Xeno
HROC29	HNPCC	wt	wt	0	0	positive	positive
HROC39	spStd	wt	wt	0	0	positive	positive
HROC40	CIMP	mt13	wt	0	0	positive	positive
HROC46	spStd	mt12	wt	1	1	***negative***	***negative***
HROC48	spMSI-H	wt	wt	0	0	positive	positive
HROC50	spMSI-H	wt	V600E	0	0	***negative***	***negative***
HROC53	spMSI-H	wt	wt	0	0	positive	positive
HROC54	CIMP	mt12	wt	2	2	positive	positive
HROC60	CIMP	wt	wt	2	2	positive	positive
HROC62	spStd	mt13	wt	0	0	positive	positive
HROC68	spStd	mt13	wt	2	2	positive	positive
HROC69	spStd	wt	wt	1	1	positive	positive
HROC71	HNPCC	mt12	wt	2	2	positive	positive
HROC80	spStd	mt12	wt	1	1	positive	positive
HROC87	spMSI-H	wt	V600E	0	0	positive	positive
HROC92	CIMP	wt	wt	2	1	***negative***	***negative***
HROC103	spStd	wt	wt	2	2	positive	positive
HROC107	spStd	mt12	wt	2	2	***negative***	***negative***
HROC112	CIMP	wt	wt	0	0	ND	positive
HROC113	HNPCC	mt12	wt	2	2	positive	positive
HROC117	CIMP	wt	wt	0	0	positive	positive
HROC123	spMSI-H	wt	V600E	0	0	positive	positive
HROC131	spMSI-H	wt	V600E	0 ^a)^	0	positive	positive
HROC135	CIMP	mt13	wt	1	1	positive	positive
HROC147	CIMP	mt12	wt	2	2	***negative***	***negative***
HROC159	spMSI-H	wt	V600E	0	0	positive	positive
HROC161	CIMP	mt12	wt	2	2	positive	positive
HROC169	CIMP	mt12	wt	0	0	positive	positive
HROC170	spMSI-H	wt	V600E	0 ^a)^	0	positive	positive
HROC171	spMSI-H	wt	V600E	0	0	positive	positive
HROC173	spStd	mt12	wt	0	0	positive	positive
HROC175	spMSI-H	wt	V600E	0	0	positive	positive
HROC183	CIMP	mt12	wt	1	1	positive	positive
HROC190	CIMP	wt	wt	2	2	positive	positive
HROC192	CIMP	wt	wt	2	2	positive	positive
HROC197	CIMP	wt	wt	2	2	positive ^b)^	positive
HROC204	spMSI-H	wt	V600E	0	0	positive	positive
HROC212	spMSI-H	wt	V600E	0	2	positive	positive
HROC213	CIMP	wt	wt	0	0	positive ^b)^	positive
HROC222	spStd	wt	wt	2	2	positive	positive
HROC250	spStd	wt	wt	0	0	positive	positive
HROC252	HNPCC	mt13	wt	2	1	positive	positive
HROC300	spStd	mt12	wt	1	1	***negative***	***negative***
HROC315	HNPCC	wt	V600E	0	0	positive	positive

^a)^ Clonally positive

^b)^ Clonally negative

SMAD4 immunohistochemistry was completely negative in six of the primaries as well as in their xenografts, whereas in 36 cases cytoplasmic and nuclear immunostaining was found in primaries and the corresponding xenografts (details in Table 2). SMAD4 immunohistochemistry of two primaries revealed an unusual “mosaic”pattern, identical in repeated reactions, but it was not found in the corresponding xenografts (example in Fig 4). Important to note, the nuclei of stromal cells in the primaries as well as in the xenografts consistently were immunopositive (Fig 4).

In our series, evaluations of pERK1/2 immunohistochemistry of the primaries turned out to be challenging. In primaries, pERK1/2 immunohistochemistry could not be evaluated with confidence because a consistently positive immunoreaction of stromal cell nuclei (as internal controls) was not observed. However, pERK1/2 immunohistochemistry worked for the xenografts. Nuclear immunostaining of stromal cells was seen in all xenografts, and tumour cell nuclei were positive in 36 of the cases (81.3%; example in S1 Fig).

The K-ras or B-raf gene mutational status did not correlate with the degree of podia formation or tumour budding in the xenografts.

## Discussion

This study addressed changes of invasion phenotypes in colorectal carcinoma xenografts as compared to their primaries. Subtle differences in the histology of primary colorectal carcinomas and their xenografts have been observed and described before, not long after introduction of the xenografting technique into experimental pathology [10]. However, at that time the phenomenon of tumour budding and podia formation and its role in surgical pathology had not been recognized, and molecular aberrations in colorectal cancers as well as details of signal transduction pathway dysregulation were entirely unknown. This prompted us to re-visit the topic for an assessment in the light of contemporary concepts. Taking advantage of the complete absence of immunoglobulins in NSG mice, the xenograft recipients in this study, we could carry out successfully our immunohistochemical reactions with exactly the same reagents and under the same conditions as used in surgical pathology. As far as we are aware this is a novel approach, and it may perhaps also be useful for other investigations into the biology of solid tumours.

We observed that median values for tumour budding and podia-formation, as determined on cytokeratin immunostains in "hot spots", were lower in xenografts than in primary colorectal carcinomas, and comparison on a case-by-case basis showed reduction in the majority of the xenografts. Thus, the xenograft microenvironment appeared to be less permissive of the aggressive invasion phenotypes of tumour budding and podia-formation, although, basically, they both are found in this context, too.

Deficient in T and B cells, NSG mice are not capable of mounting anti-tumour immunity by the adaptive immune system. Therefore, xenografting colorectal cancers into NSG mice allows study of tumour budding and podia by an approach that on one hand is analogous to that of surgical pathology, but that, on the other hand, uses a microenvironment lacking a tumour specific anti-tumour host response. In previous publications [11], it has been suggested that an anti-tumour immune response could counteract tumour budding. According to this hypothesis, tumour cells sprouting from the neoplastic glands could be particularly vulnerable to an attack by cytotoxic T cells that are part of the peritumoural lymphohistiocytic response, which often is quite brisk at the invasive margins of tumours with prominent tumour budding. Being “nipped in the bud” is the term coined for this, and this may be expected to be most efficient in microsatellite unstable tumours as they are the ones which most often are “immunoreactive” [12]. This hypothesis predicts that, by removing tumours from the restraints of the adaptive immune system, tumour budding would be more pronounced in NSG mouse xenografts than in their primaries, particularly so in the group of microsatellite unstable tumours. However, this was not seen in this series: quite to the contrary, tumour budding was observed to decrease, in the spMSI group, even to a degree that attained statistical significance (see Fig 2).

NSG mice are not devoid of myeloid cells. Although by H&E histology the peritumoural stroma in the xenografts was not appreciably infiltrated by granulocytes or macrophages, chemokines or cytokines secreted by this type of immune cells might still be present in relevant concentrations. As experimental induction of an unspecific peritumoural inflammation in subcutaneous colorectal carcinoma xenografts has a profound effect on tumour cells metamorphosing into a migratory phenotype [13], this is an important aspect. In order to explore if cytokine and/or chemokine signalling is active in our xenografts, we performed pSTAT3 immunohistochemistry because nuclear pSTAT3 immunostaining is indicative of cytokine/chemokine receptor activation. We observed complete absence of nuclear pSTAT3 immunostaining in all tumour cells of all our xenografts whereas, with the antibody cross reacting with murine pSTAT3, stromal cells as internal controls of the immunoreactions were consistently positive in all cases (Fig 4). It may be concluded that in the NSG mouse xenograft microenvironment tumour cells are completely removed from the influence of cytokine/chemokine signalling, but tumour budding and podia formation can proceed nevertheless.

Wnt, BMP and ras signalling are recognized as the three major signalling pathways prone to dysregulation in colorectal carcinoma. Dysregulation in many cases is caused by gene mutations of cardinal components (e.g. APC or β-catenin, TGFβ, and K-Ras or B-Raf genes for Wnt, BMP, and ras pathways, respectively). However, instead of an “intrinsic” dysregulation there also may be “extrinsic” pathway activation, presumably by receptor activation within the tumour microenvironment. Weather intrinsic or extrinsic, in all cases pathway activation entails nuclear translocation of β-catenin, SMAD4, or pERK1/2, which in primaries has been assayed successfully by immunohistochemistry as a “read-out” for activation in previous studies [4, 5, 7]. Since our NSG mouse xenografts allowed immunohistochemical study by exactly the same methods as that used for primaries, we set out to explore how activation of these pathways compared between primaries and their xenografts.

We observed that the patterns of β-catenin and SMAD4 immunohistochemistry in xenografts mirrored the situation in the corresponding primaries (Tab 2). By conclusion, not only dysregulation of these pathways by intrinsic activation is transferred into the xenograft, which would be expected, but the xenograft microenvironment also emulates activation of these pathways by extrinsic factors.

Unfortunately, technical limitations of the pERK1/2 immunostains of the primaries (*vide infra* for discussion of this technical issue) did not permit confident evaluations to make primary-xenografts comparisons. pERK1/2 immunohistochemistry of xenografts, however, showed that nuclear immunostaining of stromal cells as internal controls was positive in all case, and tumour cells were positive in the majority of cases (81.3%). Published studies of pERK1/2 immunohistochemistry that used colorectal carcinoma biopsies (which do not suffer the technical limitations incurred in resections) have shown that, regardless of K-Ras or B-Raf mutation, tumour cell nuclei are clearly immunopositive in 72.2% [7]. The limited conclusion of our study in this point, therefore, is that xenografting at least does not completely abrogate ras signalling, weather intrinsic or extrinsic.

This study has several limitations that must be recognized clearly. First, we did not examine the effect that differences in the extracellular matrix could have. At least in-vitro, the adhesion properties of cancer cells to different types of matrix material are known to be quite different [14]. Since the xenograft matrix is from a different species, there could have been effects on tumour budding and podia formation that is not controlled for. Second, phosphoprotein immunohistochemistry of the primaries was not evaluable with sufficient confidence to allow primary-xenograft comparisons. This is not entirely surprising and can be explained by degradation of phosphorylations which is known to begin immediately after ischemia/hypoxia and proceeds rapidly, depleting the pool of phosphorylated proteins within less than an hour [15]. In this respect, it must be remembered that even under ideal harvesting conditions, colorectal carcinomas in surgical resection specimens are subject to ischemia by ligation of the vascular pedicle before removal of the specimen, to this is added the time needed for transport of the specimen to the pathology laboratory and for handling the specimen. In our protocol-based collection [16], times needed for these steps were recorded to take between 22–232 minutes (median 53 minutes, mean 63 minutes). To this is added the process of formalin-fixation. However, contrary to the primary tumours, xenografts can be removed within minutes after sacrificing mice, and tumour tissues are much smaller, allowing a rapid fixation. Accordingly, phosphoprotein immunohistochemistry worked for the xenografts. Third, it is a limitation of this study that all xenografts were subcutaneous. Xenografting colorectal carcinomas orthotopically into the caecum or by intrasplenic or intravenous injection is well known to produce multifokal/systemic disease in the recipients at a substantial rate [10]. It may be argued, therefore, that a study of different types of xenografts would have lead to quite different results. Possibly, tumour cells might be more susceptible to transform into budding cells and/or develop podia. However, orthotopic xenografting is much more challenging technically than subcutaneous implantations, and for this reason our experience with this type of xenografts is limited so far to a few successful cases and casual observations (unpublished data). In these, a migratory phenotype was found focally in one case, but pSTAT3 immunohistochemistry with orthotopic xenografts remained completely negative in the tumour cells of all the cases, recapitulating the findings in subcutaneous xenografts.

## Conclusions

Summing up our main findings, we observed that the aggressive invasive phenotypes of tumour budding and podia formation both are found in the xenograft setting, albeit reduced quantitatively. The absence of a host anti-tumour immunresponse in the NSG mouse xenograft setting did not lead to an increase of tumour budding and/or podia formation. NSG mouse xenografts were amenable to immunohistochemical study with exactly the same methods as used for primaries. Based on these, we observed that dysregulation of wnt and BMP signalling is mirrored in the xenograft microenvironment, but cytokine/chemokine signalling apparently is not.

## Supporting information

S1 Fig Example of pERK1/2 immunohistchemistry. Note immunolabelling of some tumour cell nuclei (white arrows on exemplary nuclei) as well as stromal cells (black arrows). Image is from HROC183.
(TIF)
