# Supplementary figures and images for: Colorectal carcinoma tumour budding and podia formation in the xenograft microenvironment
Source: PLoS One. 2017 Oct 17;12(10):e0186271. doi: 10.1371/journal.pone.0186271 (PMC5645095; doi:10.1371/journal.pone.0186271)

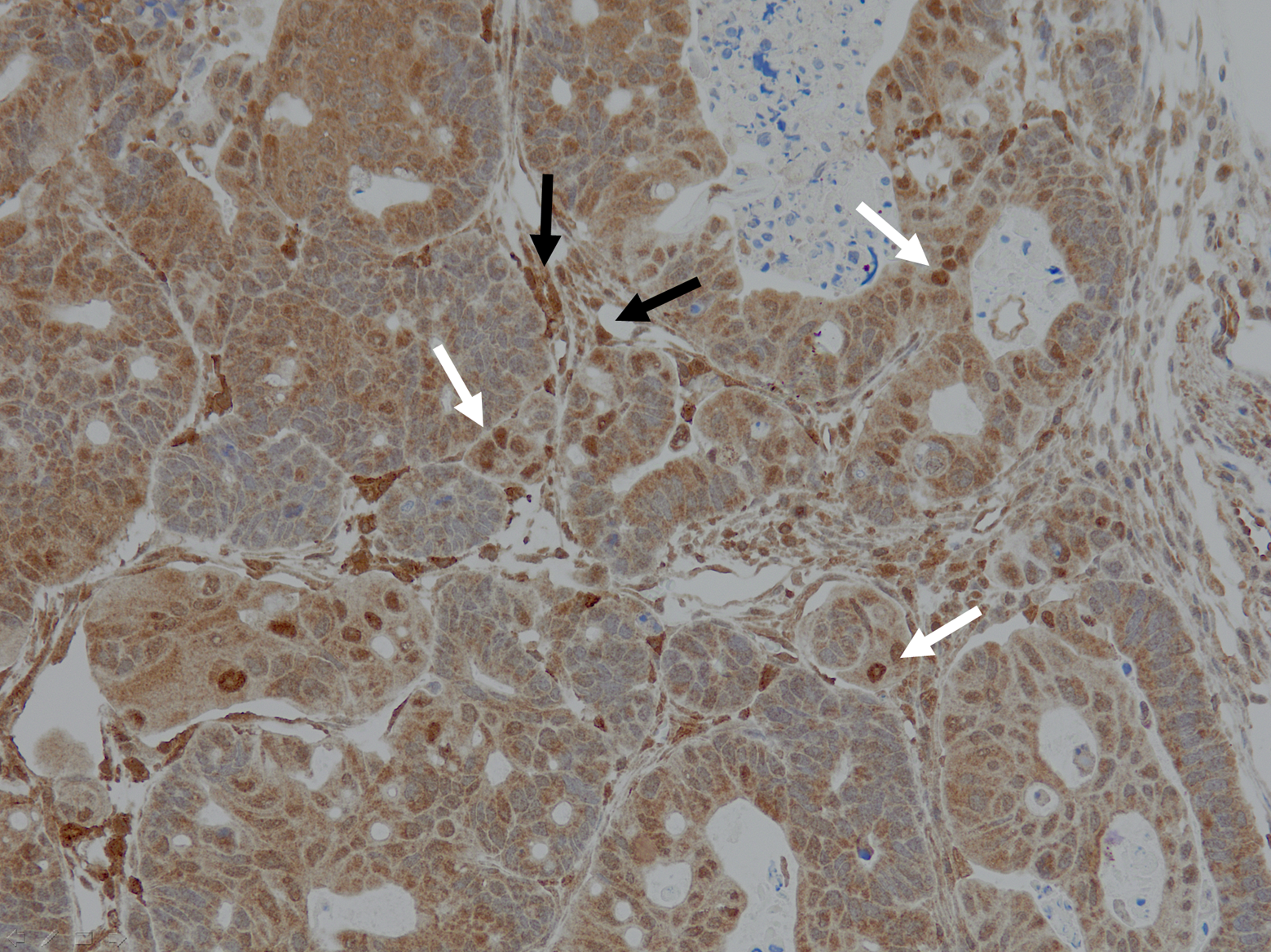

Supplement: S1 Fig — Note immunolabelling of some tumour cell nuclei (white arrows on exemplary nuclei) as well as stromal cells (black arrows). Image is from HROC183. (TIF) [file pone.0186271.s001.tif]
